# Queens become workers: pesticides alter caste differentiation in bees

**DOI:** 10.1038/srep31605

**Published:** 2016-08-17

**Authors:** Charles F. dos Santos, André L. Acosta, Andressa L. Dorneles, Patrick D. S. dos Santos, Betina Blochtein

**Affiliations:** 1 Departamento de Biodiversidade e Ecologia, Faculdade de Biociências, Pontifícia Universidade Católica do Rio Grande do Sul, Av. Ipiranga, 6681, 90619-900 Porto Alegre, RS, Brazil; 2 Departamento de Ecologia, Universidade de São Paulo, Rua do Matão, 321, Travessa 14, 05508-090 São Paulo, SP, Brazil; 3 Núcleo de Pesquisa em Biodiversidade e Computação - BioComp. Escola Politécnica, Av. Prof. Luciano Gualberto, Trav. 3, n. 380, 05508-010 São Paulo, São Paulo, Brazil; 4 Instituto do Meio Ambiente, Pontifícia Universidade Católica do Rio Grande do Sul, Av. Ipiranga, 6681, 90619-900 Porto Alegre, RS, Brazil

## Abstract

Bees are important for the world biodiversity and economy because they provide key pollination services in forests and crops. However, pesticide use in crops has adversely affected (decreased) queen production because of increased mortality among larvae. Here, we demonstrated that *in vitro*-reared queens of a neotropical social bee species (*Plebeia droryana*) also showed high larval mortality after exposure to an organophosphate pesticide (chlorpyrifos) via larval food. Moreover, most of the surviving larvae that were destined to develop into queens became workers more likely because they ate less food than expected without pesticide skewing thus caste differentiation in this bee species. This adverse effect has not been previously reported for any other social insects, such as honeybees or bumblebees. Queens are essential for breeding and colony growth. Therefore, if our data are applicable to other pantropical social bee species across the globe, it is likely that these bees are at a serious risk of failure to form new colonies.

The bee population is declining worldwide, and pesticides are among the controversial factors behind this phenomenon^1^,^2^,^3^. Although pesticides can effectively combat agricultural pest insects, they may have adverse sub-lethal effects on beneficial insects including bees compromising thus ecological service of pollination^1^,^2^,^3^. For example, pesticides can severely compromise cognition, foraging, navigation, homing, and memory abilities of honeybee and bumblebee workers^4^,^5^,^6^. Similarly, queens may also develop serious problems when exposed to pesticides: damage to ovarian tissues, high mortality, and workers’ rejection as well as difficulties with emerging, mating, and laying eggs (Supplementary Table S1).

To date, most of the toxicological research (on pesticides) has been focused on the vulnerability of honeybees and bumblebees (Supplementary Fig. S1) because they are prime pollinators in the global agriculture. Nevertheless, these bees are not always the most effective pollinators in many regions of the world^7^,^8^. For instance, on pantropical region the stingless bees play an important role as pollinators there^7^,^9^,^10^. They are ca. 45-fold and twice richer in species than honeybees and bumblebees, respectively, encompassing approximately 450 species^11^,^12^ (Supplementary Fig. S1). Only in Brazil it is believed that there are more than 300 stingless bee species^12^ which add high economic value (billions of US dollars)^13^ to crops commercialized worldwide^7^,^9^,^10^,^13^.

Stingless bees are eusocial insects forming colonies with two distinct female castes, i.e. sterile workers and reproductive queens^14^. As such, they share features observed in caste determination system of both honeybees and bumblebees as, for example, larvae destined to become queens receive/consume larger amount of larval food than those that become workers^14^,^15^,^16^. Furthermore, stingless bees (as also bumblebees) do not receive royal jelly during larval development like seen for honeybees^14^. Additionally, caste determination in stingless bees is characterised by massive differential larval nourishment^14^,^15^, except the genus *Melipona* which has an alternative pathway^17^. Therefore, female larvae of stingless bees that are destined to develop into queens are reared in larger brood cells (royal cells) and hence receive more food than female larvae that will become workers^14^,^15^. This situation most probably causes *corpora allata* to synthesise greater amounts of juvenile hormone (JH) during larval development, thereby inducing queen characteristics^18^,^19^.

At present, it is unknown whether queen larvae of stingless bees may suffer any sub-lethal effects as a result of possible exposure to pesticides. Floral resources collected by bees to rear new individuals have been found to be contaminated with numerous agrochemicals including organophosphate pesticide chlorpyrifos (CPY)^20^,^21^,^22^,^23^,^24^,^25^,^26^. (Supplementary Table S2). While adverse effects on pesticide exposed bees have repeatedly been reported^20^,^21^,^22^,^23^,^24^,^25^,^27^ it is currently unknown how queen production in stingless bees may be affected, if at all.

Currently, CPY is widely used to control agricultural pest insects worldwide^28^,^29^ (Supplementary Fig. S2). In Brazil, for example, cross-checking of adjacent crops (where CPY is recommended for one of the crops) resulted in a grave warning about potential exposure of stingless bees to CPY (Supplementary Fig. S3).

Given that CPY is indicated for several crops^28^,^29^ (see Methods) that stingless bees visit naturally^14^, then it is only logical to conduct assessments of toxicological risks for these insects. Here, we analysed possible consequences of CPY exposure during larval development of *Plebeia droryana* queens. This stingless bee species occurs in large geographic areas of Argentina, Bolivia, Brazil, and Paraguay^30^. These bees seem to be vulnerable to anthropogenic disturbances (e.g. climate change^31^,^32^) owing to the necessity to undergo a reproductive diapause during winter in these localities.

## Results

### Survival probability

**Figure 1:**
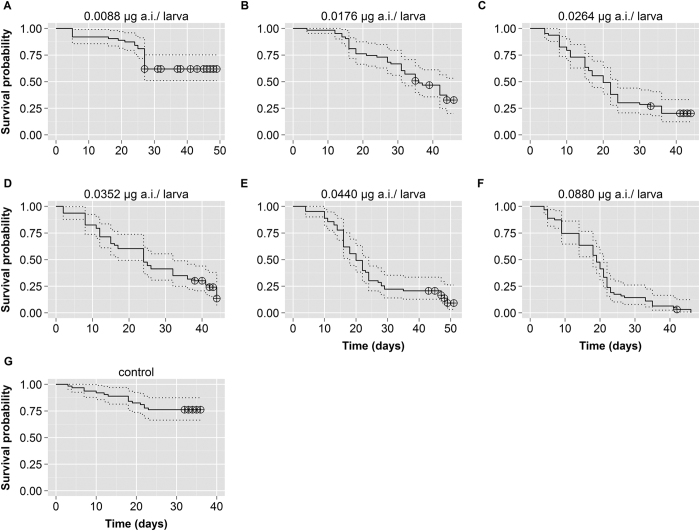
Survival probability. Six chlorpyrifos (CPY) doses (plus control) were administered to *Plebeia droryana* larvae that were fed with 66 μL of larval food and placed into a germination chamber at 25 ^o^C. Legend: full lines, survival function; dotted lines, 95% confidence interval; crossed circles, censored occurrences (emergence of bees).

We transferred 441 *P. droryana* larvae to rearing plates, all at the same temperature and humidity and with equal amounts of larval food (66 μL), but exposed to different CPY doses. Of the 441 ca. 292 individuals (larvae, pupae, or imagoes) died before emergence (Table 1). This number represents approximately 66% of the total. We also found that dead larvae or pupae darkened, starting mostly with the abdominal region (Supplementary Fig. S4). The probabilities of survival among *P. droryana* larval groups that were exposed to one of six doses of CPY and controls were significantly different (χ^2^ log-rank test = 119, degrees of freedom [d.f.] = 6, P < 0.0001; Fig. 1 and Table 1). The control treatment yielded the best survival among the larvae (76%), followed by the treatment 0.0088 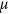
g a.i./bee (62% survival) without a significant difference between these two regimens (Bonferroni, P > 0.05, Fig. 1a,g, Supplementary Table S3). In contrast, all other treatments were significantly different from the control in terms of survival (Fig. 1b–f and Supplementary Table S3). The higher CPY dose, tested here (0.0880 μg a.i./bee) on *P. droryana* larvae, killed all treated individuals, except one that developed into a worker. We also found that only the 0.0352 μg a.i./bee treatment (48 h, 7%) and control (72 h, 1.6%) caused any larva mortality on the first 3 d of the experiment.

### Caste differentiation

Of the 441 larvae that were exposed to CPY, only 149 survived, but not all as queens as expected. That is, we found that different CPY doses in the food of *P. droryana* larvae had a significant effect on caste differentiation causing the deviation from queens to workers (generalised linear mixed model [GLMM] binomial, n = 441, groups = 48 and 7, z test = −2.59, P = 0.009). We noted that those individuals that developed into workers consumed only ca. ⅓ to ⅔ of the whole amount of larval food originally offered (66 μL) consequently they ingested lesser CPY doses than that previously provided (Supplementary Table S4).

**Figure 2:**
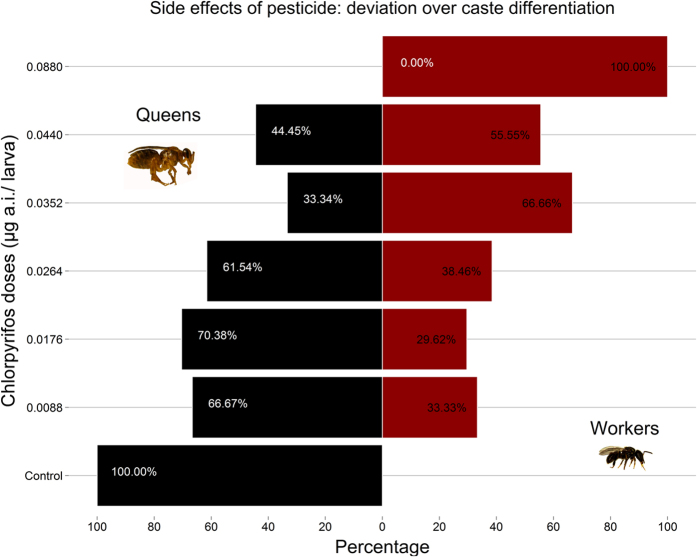
Caste differentiation. The proportion (%) of *Plebeia droryana* larvae that developed into queens is shown (all larvae were reared to become queens). Depending on the chlorpyrifos (CPY) dose in larval food, caste differentiation was skewed toward workers.

In Fig. 2 and Supplementary Fig. S5, we can see that all treatments produced statistically significant changes, except for the control. Thus, we had at least 27% of worker emergence in our experiments when 0% was expected because the amount of larval food provided was sufficient to produce only *P. droryana* queens, as observed in the control.

### Development time

**Figure 3:**
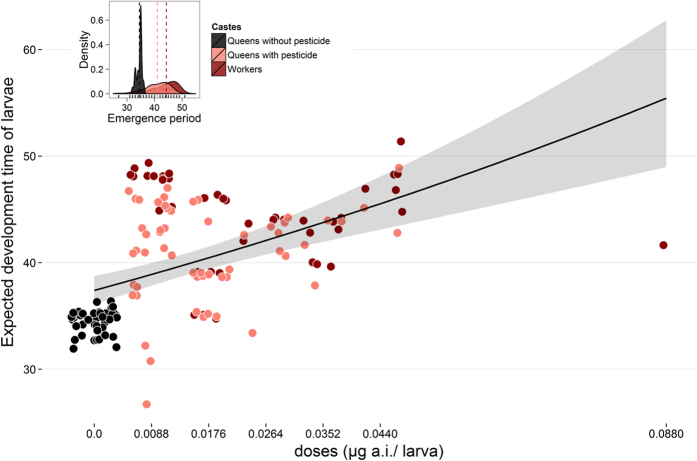
Development duration. The expected period in days for *Plebeia droryana* larvae to develop into queens and workers as a function of the chlorpyrifos (CPY) dose in larval food. The Poisson regression model (log link) is shown. Legend: dots, observed data (jitter plot); full line, predicted model; shaded area, 95% confidence interval (generalised linear mixed model [GLMM] Poisson, estimate = 4.43, standard error = 1.29, z-value = 3.4, P < 0.001); the smaller chart, density; dotted lines, means.

We found that the duration of development of *P. droryana* larvae was also strongly affected by CPY in larval food (GLMM Poisson, n = 441, groups = 2, z test = 3.41, P < 0.001; Fig. 3). Overall, control queens developed faster (34 ± 1.01 d, mean ± standard deviation [SD]) than did the CPY-exposed queens (41 ± 4.45 d, mean ± SD) and CPY-exposed workers (44 ± 4.29 d, mean ± SD), all of which received various doses of the pesticide with larval food (χ^2^ = 83.95, d.f. = 2, P < 0.001; Fig. 3, smaller chart and Supplementary Table S5). Therefore, our *P. droryana* larvae (originally reared to emerge as normal queens) took 1.2-fold more time (CPY-exposed queens) and 1.29-fold more time (CPY-exposed workers) to develop than did control queens.

## Discussion

Our results point to two important findings related to stingless bee larvae and their vulnerability to CPY. Firstly, the greater the pesticide concentration in the larval food of *P. droryana*, the lower was the queen emergence rate because of deaths during the period of larval development. Most of those dead larvae, pupae, or imagoes had severe deformities resembling those observed in *Melipona quadrifasciata* worker larvae treated with biopesticides azadirachtin and spinosad^33^. Secondly, with the increasing pesticide dose in the larval food, there was a greater chance for would-be queens to become workers.

The high mortality rate of *P. droryana* queen larvae that were treated with various CPY doses could be expected, as similar results were observed in the queen larvae of honeybees^27^; however, the differentiation of many surviving larvae into the worker caste rather than queens was not expected. This effect is most probably mediated because the caste differentiation system found in most genera of stingless bees^14^,^15^,^18^,^19^. In other words, those *P. droryana* bees that emerged as workers consumed less larval food (ca. **⅓**–⅔) and this alteration prevented them from acquiring queen attributes such as a larger size, the absence of corbiculae, and the presence of spermathecae. In fact, we could observe larger waste food in treatments resulting in workers than in queens, albeit we did not have estimated how much was remained there. At present, we have data that some bees (*Apis mellifera* and *Bombus terrestris* workers) do not avoid contaminated food as they find it attractive^34^. However, this may be different for different pesticides and also in different context.

At lethal doses, CPY inhibits the breakdown of acetylcholine by irreversibly binding to the active site of cholinesterase; the build-up of acetylcholine overstimulates neuronal cells, thus causing neurotoxicity and leading to the insect’s death^35^,^36^. At sub-lethal doses, however, CPY’s physiological effects on bees are still poorly understood. Therefore, we believe that in future studies, researchers should test whether *P. droryana* larvae that are destined to become queens consume less food under these conditions because this food is less palatable or then CPY acts on their central nervous system or digestive tract.

Larval development time of *P. droryana* was also significantly affected by CPY. Although control queens developed within the expected period (~35 d^37^), the corresponding CPY-exposed individuals took more time to develop into queens or workers. The differentiation into queens (in controls) that we observed here may be explained by the regular intake of larval food, which likely causes greater production of JH during larval development, thus inducing the proper caste differentiation^18^,^19^. Nonetheless, it is unclear why some CPY-exposed larvae still emerged as queens, even though taking significantly more time to develop and consequently being more exposed to CPY doses. Nor could we determine whether the larvae consuming smaller amounts of larval food and emerging as workers could not synthesise and utilise a sufficient JH titre in order to differentiate into queens. This issue needs further research in order to evaluate whether such larvae could use a part of energy obtained from food to metabolise (i.e. detoxify) CPY rather than sustain proper growth and development.

It is known that would-be queens among *P. droryana* larvae consume c.a. sevenfold more larval food than regular workers do^37^. Even though we expected that the *P. droryana* larvae consuming smaller amounts of larval food would develop into workers, we also expected to see some miniature queens. It is believed that under some conditions, female larvae of social insects may self-determine their own developmental fate^38^. For example, it has been postulated that stingless bee larvae reared in brood cells and destined to become workers (hence, receiving smaller nourishment) could adopt a selfish strategy by evading the worker fate and developing into dwarf queens^39^,^40^. Such alternative queen phenotype found in some social insects including stingless bees may naturally head new colonies with relative reproductive success, albeit its fecundity after mating be still few comprehended^39^,^40^. Nevertheless, most of the malnourished *P. droryana* larvae observed here became workers (except two intercaste individuals with worker size but having corbiculae and spermatheca) rather than dwarf queens. Therefore, if *P. droryana* larvae that were fated to develop into workers can make this ‘choice’, then exposure to CPY appears to impair this self-determination ability.

The adverse effect of CPY, i.e. the significant skew in caste differentiation of *P. droryana* from queens to workers, was a surprise because we expected to see increased larval mortality, as is the case for *A. mellifera* queen larvae that are treated with CPY^27^. Therefore, if our data are applicable to other stingless bee species inhabiting other regions of the world (Supplementary Fig. S2) – whose caste determination mechanism is similar to that of *P. droryana* – then viability of new queens may be seriously jeopardised. As mentioned above, CPY has been indicated for many crops^28^,^29^ typically cultivated in Brazil (Supplementary Fig. S3) and many other countries where stingless bees occur naturally. Furthermore, traces of CPY can in fact be found in pollen grains and nectar, sometimes as a major contaminant inside colonies (e.g. in stored pollen, honey, or beeswax)^20^,^21^,^22^,^23^,^24^,^25^,^26^ beyond a myriad of other pesticides like neonicotinoids^23^,^24^,^25^,^26^,^41^,^42^,^43^,^44^,^45^,^46^,^47^.

Various research groups have detected CPY in pollen grains at a concentration that exceeds the doses tested here, which caused larval death (Supplementary Table S2). These observations support a possible scenario where stingless bee populations may have difficulty producing new queens if similar CPY levels are chronically received by colonies via pollen and nectar and then are provided via larval food to queens. Because stingless bee queens are indispensable for breeding and colony growth, the abnormal conversion of queens into workers may seriously compromise the successful use of these bees for the purposes of agricultural pollination, for example, as often proposed^7^,^9^,^13^. This data is still more troublesome because overall queen production in stingless bees is naturally limited to a few individuals per year, lesser than 1–2% of the whole colony population (except for the *Melipona* genus)^15^. Therefore, it is necessary to extend such experiments to other species of stingless bees.

We are aware that toxicity bioassays, such as the one presented here, may not accurately reflect the pesticide concentrations found under real-life conditions where other climatic and ecological variables may affect the pesticide levels^48^. This is a limitation of our study. Additionally, we are not discussing and explaining the important role that pesticides overall have played in the effective control of thousands of agricultural pests that annually cause high economic losses^28^,^49^. Rather, alternative methods of pest control involving the selective use of pesticides in crops may help to reduce toxic exposure among these beneficial insects, which are responsible for pollination of many wild and cultivated plants^7^,^8^,^9^.

In summary, pesticide exposure of stingless bees, which are important pantropical pollinators, is still a neglected topic of research^50^. Here, we demonstrated that exposure to different pesticide doses (CPY) may significantly alter the expected production of queens toward workers even if larvae consume just part of the amount of the provided contaminated food with such residuals. It may put at risk the growth and maintenance of natural populations of stingless bees. Although similar losses in queen production have been observed in honeybees and bumblebees (Table S1), the skewed caste determination under the influence of a pesticide seems to be specific to stingless bees. Long-term studies will help us to evaluate the adverse effects of such pesticides on the adult population’s survival and on viability of this stingless bee species (*P. droryana*).

## Methods

### Queen rearing and toxicological assays

*In vitro* queen-rearing of *P. droryana* was based on a protocol developed for this species^37^. Thus, all main steps that were used here can be found in that protocol^37^, from collection of larvae to harvesting of larval food. In the present study, we used three to five colonies of *P. droryana* (depending on its internal state) from a stingless-bee apiary of the School of Biosciences, Pontifical Catholic University of Rio Grande do Sul, Brazil.

Larval food containing nourishment sufficient to produce 21 *P. droryana* queens per treatment – every larva consumed 66 *μ*L of larval food during its development – was stored in Eppendorf tubes until preparation of CPY doses.

First, a stock solution of CPY (Lorsban^®^ 480BR, 48% a.i., Dow AgroSciences, Brazil) was prepared at 1 *μ*g a.i./*μ*L in distilled water. Next, we mixed the stock solution of CPY with larval food to prepare the following doses: 0.0088, 0.0176, 0.0264, 0.0352, 0.0440, and 0.0880 *μ*L a.i./bee as well as a control, i.e. food without the pesticide. These concentrations were selected after we evaluated median values of CPY concentration in pollen grains according to the literature (Supplementary Table S3, mainly references 7 and 8). Then, we chose the concentrations that would be close to sub-lethal for *P. droryana* larvae in our experiments, by taking into account the amount of pollen consumed by a larva during its development^51^ (ca. 91,000 *μ*g, i.e. seven- to eightfold more than the amount for a prospective worker larva)^37^.

This larval food (i.e. treatments: six CPY doses as well as the control) was transferred into separate rearing plates. After that, we placed *P. droryana* larvae on top of the food (one per cavity). Finally, these queen-rearing plates were placed in hermetic plastic containers (7 × 11 × 17 cm) and were kept in an incubator at 25 °C (model Luca-161/04, LUCADEMA, Sao Paulo, Brazil), in constant darkness (0L:24D) throughout the whole experiment. The humidity was controlled as described by Santos *et al*.^37^. Next, we daily monitored larvae, pupae, and imagoes for signs of imminent death: a darkened tegument. Only the immatures that were fully dark were removed from the experiments and considered dead.

Every treatment series was performed in triplicate, that is, 21 larvae per plate × seven treatments (six doses plus control) × three replicates, totalling 441 tested larvae at the end of the experiment.

### Survival analysis

To evaluate the number of larval deaths and emergence of adult individuals as a function of time, we analysed the survival probability of *P. droryana* larvae at different pesticide doses (treatments). For this purpose, we carried out the Kaplan-Meier survival analysis. Then, we tested the data for differences between survival curves using the G-rho family of analyses (here, χ^2^ log-rank test), assigning equal weight to each time point estimate. Next, we conducted multiple pairwise comparisons between treatments using the Bonferroni-adjusted method as the family-wise error rate (FWER). These data were analysed by means of the ggsurv function of the *GGally*^52^ package and by means of the *survival*^53^, *KMsurv*^54^, and *scales*^55^ packages of the R software.

### Caste differentiation and larval development

We wanted to find out whether various pesticide doses would have any effect on the probability of larvae becoming workers since these larvae received the same amount of larval food (66 *μ*L) suitable for development into queens. Due to that, we verified that those individuals that developed into worker bees consumed ⅓ to ⅔ of the initially provided larval food after visually comparing the proportion of waste food left unconsumed. Next, we estimated the mean and standard deviation of larval food within this range as well as CPY doses concerning every treatment. For this, we applied inferential statistics using the *runif* function in R to generate random deviates on the interval from ⅓ to ⅔ of larval food and CPY doses taking into account the number of workers emerged in each treatment. Then, we bootstrapped these values and replicated them 5,000 times using the *sample* and *replicates* functions in R.

We also wanted to evaluate the effects of the pesticide on the duration of larvae development. In this regard, it is known that the development time of queens is shorter than that of workers. Thus, we analysed these data using the GLMM because we needed multiple repeated-measures analyses across time (longitudinal data) for each treatment. In addition, this method could take into account possible variation among random-effect predictors^56^,^57^. In the first model, we evaluated the probability that the *P. droryana* larvae that were destined to become queens would become workers (no = 0; yes = 1) by assuming that the treatments were fixed-effect predictors and that the development duration and dose repetitions were random-effect predictors. Such a model was fitted to a binomial distribution (logit) for binary data.

In the second model, we tested whether the pesticide doses (treatments = fixed effects) had an effect on the duration of development of *P. droryana* larvae into queens or workers. Here, we assumed that dose repetitions were random effects and that the status (queen or worker) was their covariate. In this case, we used Poisson distribution errors again by means of the function *lme4* package^58^. Both models were adjusted by means of maximum likelihood (Laplace approximation).

These models were also subjected to bound optimisation by quadratic approximation (“bobyqa”), an algorithm for estimation of variance-covariance matrices of random effects. These approaches were implemented by means of the function glmer from the *lme4*^58^ package. Each model was then analysed for overdispersion data using the function overdisp.glmer from the *RVAideMemoire*^59^ package. Significance was tested by the χ^2^ test as follows: 1 – pchisq (residual deviance/d.f.). There was no overdispersion in the data (χ^2^, ratio: 0.166 and 0.192, P > 0.05, for the first and second model, respectively); this finding showed that binomial and Poisson error distributions were adequate. All statistical analyses were carried out in the R software^60^.

Finally, we also compared the development duration of workers and queens after evaluating normality by the Shapiro-Wilk test. Then, we conducted a Kruskal-Wallis analysis, followed by Dunn’s test for multiple comparisons adjusted by the Benjamini-Hochberg method (from the *dunn.test*^61^ package).

### Distinguishing queens from workers

Morphological discrimination of castes among adult bees of some species is possible, for example, by the presence or absence of pollen-carrying and pollen manipulation structures on the third leg pair (tibial corbicula, rastellum, and auricle). The bee taxon under study is known as corbiculate bees^62^ where we can find closely related tribes such as honeybees (Apini), stingless bees (Meliponini), bumblebees (Bombini), and orchid bees (Euglossini)^63^. The first two are considered highly eusocial bees. Thus, in stingless bees, only workers have the corbiculae in order to manipulate and carry on only pollen but also resin, wax, mud, and even seeds^14^. Therefore, to accurately identify the emerging individuals in our groups of *P. droryana*, we individually examined the specimens for the presence or absence of the corbicula (Supplementary Fig. S6). The corbicula-containing individuals were assumed to be workers. Moreover, we performed an additional test by dissecting the abdomen of all these bees in order to determine whether they also had a spermatheca (Supplementary Fig. S7). This structure helped us to reliably identify the *P. droryana* queens because only this caste has this structure (for storage of spermatozoa after mating).

### Worldwide scale map

Here, we estimated the potential vulnerability of stingless bees to chlorpyrifos on countries where they naturally occur and where there is evidences for chlorpyrifos usage. For this, we surveyed the georeferenced stingless bee occurrences from the Global Biodiversity Information Facility (GBIF, 2015, www.gbif.org). Then, we superimposed the occurrence points on the polygons (shapefile format) of “countries administrative area” obtained from Global Administrative Area (GADM, 2015, version 2.0; www.gadm.org). The generated map was performed in the Geographic Information Systems ArcGIS software, version 9.3, by means of the “select by location” function (ESRI Inc., 2010, www.esri.com). After that, exclusively for the countries whose administrative areas are overlapped with natural habitats of stingless bees, we searched by official trade representatives on the World Wide Web for reliable evidence of the use or commercialisation of at least one brand name of insecticides containing CPY (0,0-diethyl-0-3,5,6-trichloro-2-pyridyl phosphorothioate).

We assumed that this evidence means the presence of CPY in crops in a country, consequently indicating that stingless-bee species in this country were likely to come into contact with this insecticide (= potential vulnerability). Using these data, we built a world map (Supplementary Fig. S2) exhibiting the risk of stingless bees’ being exposed to CPY.

### Researches regarding the problem of bees and pesticides

There are evidences for significant gaps in the knowledge about the interactions of pesticides (any one) and native, wild bees^50^. Based on this, we did a search in the Web of Science™ database using the terms bees* AND pesticides* and linking these words to Topic in order to find articles and studies dealing with these issues. The search was limited to the period from January 01, 2014, to September 26, 2015.

Here, we were interested in all scientific publications: from research papers or state-of-the-art reviews to commentaries and other articles related to bioassays, experiments, discussions, and opinions on any kinds of pesticides and their effects on bees (larvae or adults; workers or queens, where applicable). We found 217 articles dealing with the topic at hand (bees* AND pesticides*). Then, we analysed the whole titles, abstracts, main text, and materials and methods sections (where applicable) of these articles to find out which bee species were studied, evaluated, mentioned, or discussed in relation to pesticides.

During this search, we encountered bee species that could be separated into four large groups: honeybees (Apini: *Apis mellifera, Apis cerana*, and other *Apis* spp.), bumblebees (Bombini: *Bombus terrestris, Bombus impatiens*, and other *Bombus* spp.), stingless bees (Meliponini: e.g. *Scaptotrigona* aff. *depilis* and *Melipona quadrifasciata*), and wild or solitary bees (e.g. *Megachile rotundata* and *Osmia lignaria*). We then found out whether there was greater interest in or a study on a specific bee group. For this purpose, we carried out the Shapiro-Wilk normality test, followed by the Kruskal-Wallis rank sum test with multiple pairwise comparisons computed by Dunn’s test from the *dunn.test*^61^ package. This analysis was adjusted by the Benjamini-Hochberg method for control of the false discovery rate.

### Brazil scale map

Considering that our case study was conducted in Brazil, we estimated and mapped the potential vulnerability of stingless bees to CPY by Brazilian municipalities. To this end, we obtained from the Brazilian Institute of Geography and Statistics (IBGE, 2015) the data on the average annual harvested area (ha: hectare) for the period 2010 to 2013 by Brazilian municipalities for crops where Lorsban^®^ (480BR, 48% a.i., 0,0-diethyl-0-3,5,6-trichloro-2-pyridyl phosphorothioate; Dow AgroSciences, Brazil) had been recommended for pest control. We chose the following crops: apple, barley, citrus, coffee, cotton, maize, potato, sorghum, soybean, tomato, and wheat.

These data were superimposed onto polygons of a Brazilian municipality’s administrative area in ha; source of shapefile: IBGE, 2010) to map the crop production across Brazil. After that, we summed all crops’ harvested area averages by municipality (municipality 1: crop 1 area + crop 2 area and so on). Thus, we calculated the proportion of crop’s sampled area in a municipality area and projected these data onto the map. This procedure was again carried out into ArcGIS (version 9.3; ESRI Inc., 2010; www.esri.com) to generate the map depicted on Fig. S3.

We assumed that a greater ratio (percentage) of the sampled area to a municipality’s area meant greater vulnerability of stingless bees (occurring there) to CPY. The vulnerability level depends on the amount of the insecticide used (in litres; Lorsban^®^ 480BR, 48% a.i., Dow AgroSciences, Brazil), which is directly linked to the crop area, i.e., area size/litters. Consequently, the probability of stingless bees’ being affected by CPY increased with the increasing ratio sampled/municipality area.

## Additional Information

**How to cite this article**: dos Santos, C. F. *et al*. Queens become workers: pesticides alter caste differentiation in bees. *Sci. Rep.* **6**, 31605; doi: 10.1038/srep31605 (2016).

## Supplementary Material

Supplementary Information

## Figures and Tables

**Table 1:** The results of the GLMM for factors (chlorpyrifos doses) affecting survival probability of bees and its skewing in caste differentiation.

Treament (μg a.i./bee)	Survival probability plus 95% CI	Dead	Live			*Total*	GLMM parameters			
			Workers	Intercastes	Queens		Estimate	Std. Error	z value	Pr(>|z|)
control	0.76 [0.664–0.875]	15	0	0	48	63	−0.3125	2.1684	−0.144	0.885414
0.0088	0.62 [0.510–0.751]	24	13	0	26	63	13.2258	3.4019	3.888	0.000101
0.0176	0.32 [0.201–0.532]	36	8	0	19	63	11.7151	4.1211	2.843	0.004473
0.0264	0.20 [0.123–0.332]	50	5	1	7	63	10.6836	3.1231	3.421	0.000624
0.0352	0.13 [0.064–0.281]	51	8	0	4	63	5.6065	2.8915	1.939	0.048510
0.044	0.09 [0.031–0.262]	54	5	1	3	63	10.5075	3.0898	3.401	0.000672
0.088	0.00 [0.008–0.012]	62	1	0	0	63	8.2827	2.4862	3.331	0.000864
	*Total*	292	40	2	107	441				

a.i./bee: active ingredient per bee; 95% CI: 95% confidence interval; Dead: amount of immature bees (mostly larvae) that did die (i.e. not emerged) per treatment; Live: amount of bees that did emerge per treatment and its respective caste after we did perform a morphological analysis for presence/absence of spermatheca and corbiculae; Total: total number of larvae transferred for rearing plates per treatment; Std. Error: Standard Error; z-value: Standard score, i.e. standard deviations from their means. Negative values when raw score is below the mean, positive when above; Pr(>|z|): probability to find z-scores by chance.
