# Supplementary material for: Queens become workers: pesticides alter caste differentiation in bees
Source: Sci Rep. 2016 Aug 17;6:31605. doi: 10.1038/srep31605 (PMC4987680; doi:10.1038/srep31605)
Supplement: Supplementary Information [file srep31605-s1.pdf]

Supplementary Information

Queens become workers: pesticides alter caste differentiation in bees

Charles F. dos Santos[1]*, André L. Acosta[2,3], Andressa L. Dorneles[1], Patrick D. S. dos Santos[1], Betina Blochtein[1,4]‡

[1]Departamento de Biodiversidade e Ecologia, Faculdade de Biociências, Pontifícia Universidade Católica do Rio Grande do Sul, Av. Ipiranga, 6681, 90619-900, Porto Alegre, RS, Brazil; [2]Departamento de Ecologia, Universidade de São Paulo, Rua do Matão, 321, Travessa 14, 05508-090 São Paulo, SP, Brazil. [3]Núcleo de Pesquisa em Biodiversidade e Computação - BioComp. Escola Politécnica, Av. Prof. Luciano Gualberto, Trav. 3, n. 380, 05508-010 São Paulo, São Paulo, Brazil; [4]Instituto do Meio Ambiente, Pontifícia Universidade Católica do Rio Grande do Sul, Av. Ipiranga, 6681, 90619-900, Porto Alegre, RS, Brazil.

Correspondence to: *charles.santos@pucrs.br, ‡betinabl@pucrs.br

Supplementary Table S1. Lethal and sub-lethal effects of xenobiotics on queen bees. † – Pesticide or fungicide (Pristine®) alones or both them; ‡ – Deformed wing virus; Ω – Pesticides plus parasites (*Crithidia bombi*); Φ – Control of fungal diseases on plants; NA – Not applicable.

Sociality level	Bee species	Classes	Pesticides (common names)	Target tissue or organ in insects	Lethal and sublethal effects	Ref.
Highly eusocial bees	*Apis mellifera*	Carbamate; Benzamide	Fenoxycarb; Diflubenzuron	Mimics juvenile hormone; inhibits the production of chitin; interferes with larval molting	Fault mating and affected egglaying	[1]
		Neonicotinoid	Imidacloprid	Neurotoxin which act on the central nervous system	Mortality in the midgut columnar cells	[2]
		Neonicotinoid	Thiamethoxam, Clothianidin	Neurotoxin which act on the central nervous system	Higher queen supersedure	[3]
		Organophosphate	Chlorpyrifos	Neurotoxic for insects by inhibiting acetylcholinesterase	Mortality during pupate or pupae phases	[4†]
		Organophosphate	Chlorpyrifos	Neurotoxic for insects by inhibiting acetylcholinesterase	Higher susceptibility to viruses‡	[4†]
	Plebeia droryana	Organophosphate	Chlorpyrifos	Neurotoxic for insects by inhibiting acetylcholinesterase	Higher mortality queen larvae	Here
		Organophosphate	Chlorpyrifos	Neurotoxic for insects by inhibiting acetylcholinesterase	Skewed caste differentiation	Here
Primitively eusocial bees						
	Bombus terrestris	Neonicotinoid	Imidacloprid	Neurotoxin which act on the central nervous system	Reduced queen production	[5]
		Neonicotinoid	Imidacloprid	Neurotoxin which act on the central nervous system	Decreasing brood production	[6]
		Neonicotinoid	Thiamethoxam, ClothianidinΩ	Neurotoxin which act on the central nervous system	Low survival	[7]
	Bombus impatiens	Neonicotinoid	Thiamethoxam, Clothianidin	Neurotoxin which act on the central nervous system	Higher mortality	[8]
		NA	ChlorothalonilΦ	NA	Smaller queens	[9]

Supplementary Table S2. Chlorpyrifos traces that were found in floral resources. Available data only from the pollen and nectar harvested and/or stored by honeybees and bumblebees. N = sample size; All measures of dispersion in μg/μL.

N	Positive samples (frequency)	Min.	Max.	Median	Mean	SD or SE	References
**BEES**							
--	--	--	5.7E-05	--	--	--	10
140	12 (8.6%)	1.0E-06	1.1E-05	2.2E-06	3.4E-06	--	11
--	--	--	8.1E-05	--	--	2.7E-05 (SE)	4
--	--	--	7.3E-05	--	--	3.3E-05 (SE)	4
16	1 (6.2%)	3.3E-05	3.3E-05	3.3E-05	3.3E-05	--	12
18	1 (5.5%)	3.1E-05	3.1E-05	3.1E-05	3.1E-05	--	12
**POLLEN**							
N	Positive samples (frequency)	Min.	Max.	Median	Mean	SD or SE	References
448	10 (2.2%)	3.0E-06	2.9E-04	--	9.5E-05	--	13
397	10 (2.5%)	4.0E-06	2.8E-04	--	8.7E-05	--	13
92	6 (6.5%)	4.0E-06	1.8E-04	--	3.3E-05	--	13
84	4 (4.8%)	5.0E-06	8.7E-05	--	3.4E-05	--	13
--	--	--	8.3E-04	--	--	--	10
350	153 (43.7%)	1.0E-07	8.3E-04	4.4E-06	5.3E-05	--	11
198	1 (0.5%)	3.5E-05	3.5E-05	--	3.5E-05	--	14
--	--	--	9.7E-04	--	--	1.2E-05 (SE)	4
--	--	--	9.4E-04	--	--	3.5E-05 (SE)	4
--	7	--	1.6E-05	--	3.1E-06	1.1E-06 (SE)	15
313	14 (4.50%)	2.0E-06	2.5E-05	4.4E-06	6.8E-06	6.2E-06 (SD)	16
14	1 (7.1%)	2.4E-05	2.4E-05	2.4E-05	2.4E-05	--	12
17	11 (64.7%)	4.1E-06	7.2E-05	1.3E-05	2.6E-05	--	12
**HONEY**							
N	Positive samples (frequency)	Min.	Max.	Median	Mean	SD or SE	References
--	--	--	1.5E-05	--	--	--	10
31	13 (41.9%)	3.0E-05	8.0E-05	--	4.6E-05	1.2E-05 (SD)	17
20	1 (5%)	3.3E-06	3.3E-06	3.3E-06	3.3E-06	--	12
**BEEWAX**							
N	Positive samples (frequency)	Min.	Max.	Median	Mean	SD or SE	References
47	3 (7.3%)	7.1E-06	1.9E-05	--	1.5E-05	--	18
--	--	--	8.9E-04	--	--	--	10
258	163 (63.2%)	1.0E-06	8.9E-04	4.3E-06	2.5E-05	--	11
87	3 (3.5%)	7.1E-06	1.9E-05	--	1.5E-05	--	14
13	8 (62%)	3.0E-06	1.5E-05	--	8.0E-06	--	19

Supplementary Table S3. Log-rank test & Bonferroni adjusted results. *P*-value adjustment method for pairwise comparisons: Bonferroni

Log-rank Test

survdiff(formula = Surv(Time, Occurrence) ~ Treatments, data = Larvae.Survival.Total, rho = 0)

χ^2= 119, d.f. = 6, P < 0.001

	N	Observed	Expected	(O-E)^2/E	(O-E)^2/V
Treatments=0.0088	63	24	58.3	20.20	27.10
Treatments=0.0176	63	36	49.6	3.75	4.73
Treatments=0.0264	63	50	33.5	8.18	9.65
Treatments=0.0352	63	51	38.9	3.74	4.51
Treatments=0.0440	63	54	37.4	7.32	8.91
Treatments=0.0880	63	62	26.8	46.19	53.76
Treatments=control	63	15	47.4	22.14	28.18

Pairwise comparisons using *t* tests with pooled SD

data: Occurrence and Treatments

	0.0088	0.0176	0.0264	0.0352	0.0440	0.0880
0.0176	0.1734	-	-	-	-	-
0.0264	3.6e-07	0.0439	-	-	-	-
0.0352	1.0e-07	0.0207	1.0000	-	-	-
0.0440	2.1e-09	0.0017	1.0000	1.0000	-	-
0.0880	1.3e-14	3.6e-07	0.1734	0.3235	1.0000	-
control	0.9913	9.5e-05	1.5e-12	3.2e-13	2.6e-15	< 2e-16

Supplementary Table S4. Quantitative and qualitative factors causing failure in queen production due to exposure to pesticides. Estimated amount of larval food consumed and subsequent expected amount of chlorpyrifos residuals ingested by would-be queen larvae becoming workers.

**Amount of larval food offered**	**Estimated food range consumed ⅓ to ⅔**		**Mean ± Std.dev.**
66µL	22µL	44µL	31.55µL ±0.97
**Treatments** (µg a.i./ larva)	**Estimated dose range from ⅓ to ⅔** (µg a.i./ larva)		**Mean ± Std.dev.** (µg a.i./ larva)
0.0088	0.0029	0.00587	0.003876 ±1.80e-04
0.0176	0.0059	0.01173	0.008853 ±6.30e-04
0.0264	0.0088	0.0176	0.01373 ±8.400e-04
0.0352	0.0117	0.02347	0.01721 ±1.260e-03
0.0440	0.0147	0.02933	0.02525 ±1.590e-03
0.0880	0.0293	0.05867	0.04077 ±0.000

Supplementary Table S5. Development duration. The Kruskal-Wallis test for multiple comparisons among bees yielded "normal" queens (control) and chlorpyrifos-exposed queens and workers (pesticide bioassays). Note: *P*-value adjustment method for pairwise comparisons: Benjamini-Hochberg; Bold numbers indicate the test value; Asterisks indicate statistically significant at 0.001 level.

**Kruskal-Wallis:** χ^2= 83.9584, d.f. = 2, P < 0.001		
	contaminated worker	contaminated queens
contaminated queens	**2.988602**	
	0.0014*	
control queens	**8.897366**	**6.417743**
	0.0000*	0.0000*

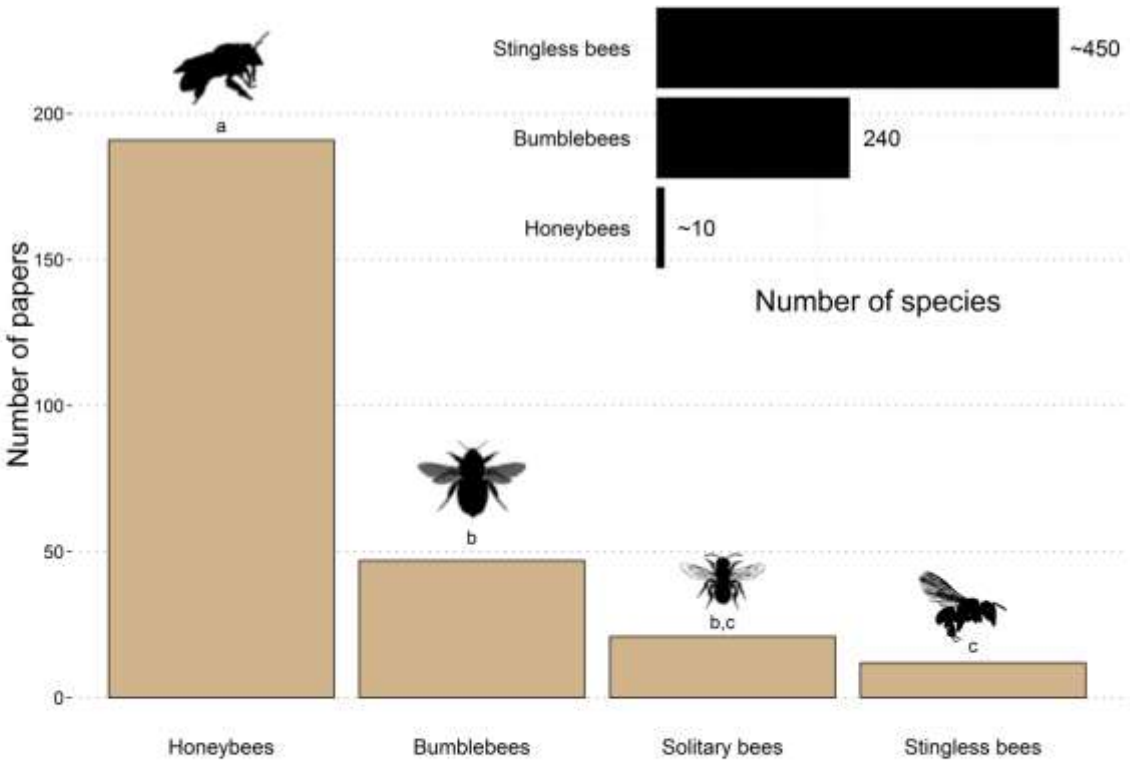

Supplementary Figure S1. Research efforts and/or scientific concerns regarding pesticide effects on bees. Source: Web of Science™ (January 01, 2014, to September 26, 2015). Kruskal-Wallis, $\chi^2 = 117.8$, d.f. = 3, $P < 0.001$. Different letters mean a significant difference according to *post hoc* Dunn's test with the Benjamini-Hochberg adjustment. Smaller chart: Amount of eusocial bee species. Note: The bumblebee silhouette was drawn by Melissa Broussard which is shared via PhyloPic project (www.phylopic.org). It is available for use/reuse under the Creative Commons Attribution-ShareAlike 3.0 Unported license (http://creativecommons.org/licenses/by/3.0/). Honeybee and solitary bee silhouettes are available for use/reuse under the Public Domain Dedication 1.0 license (retrieved from www.phylopic.org); Stingless bee silhouette from author's archive.

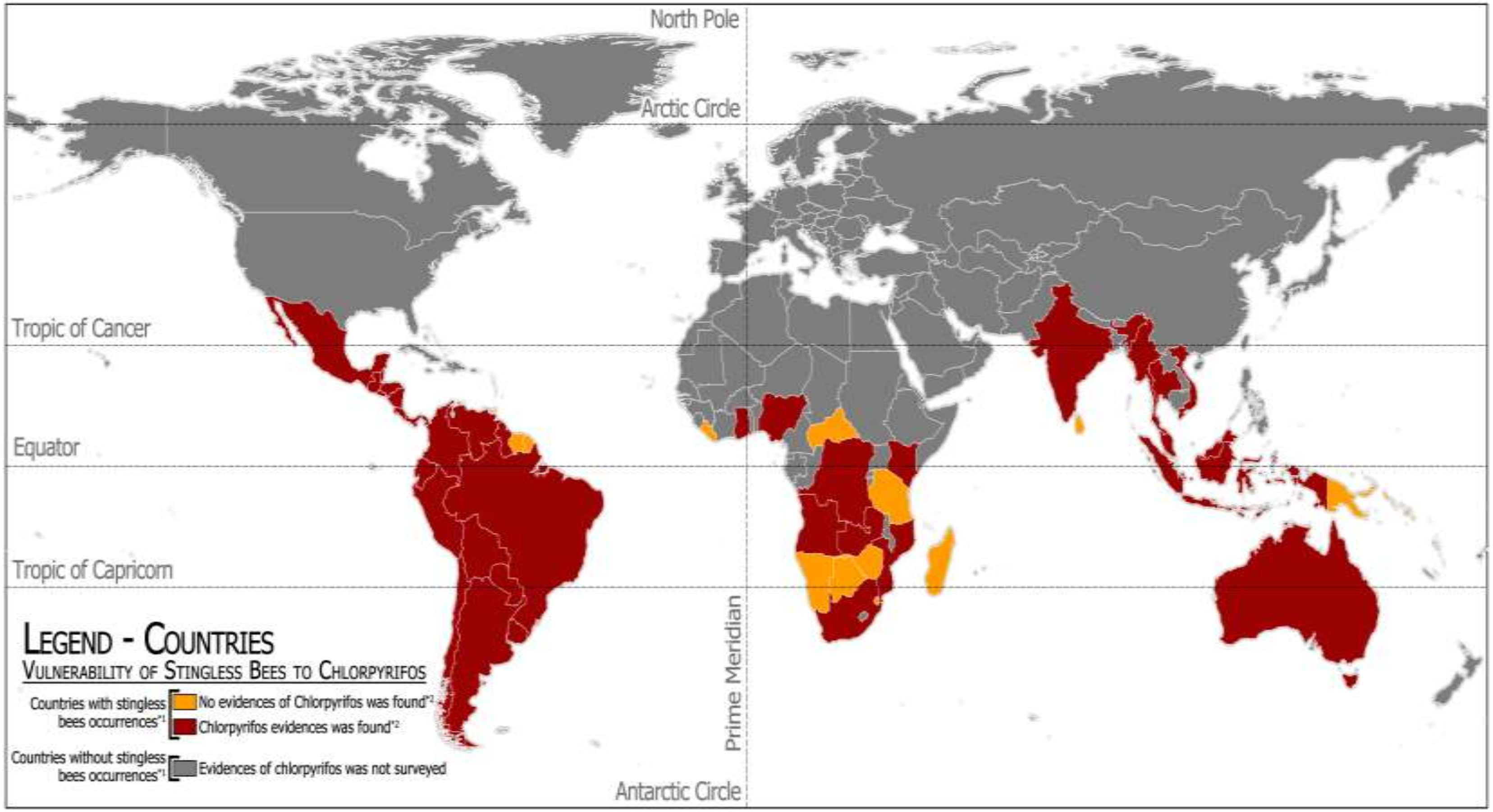

Supplementary Figure S2. Countries with possible vulnerability of stingless bee populations to chlorpyrifos (CPY). Data were plotted according to natural occurrence of stingless bees across countries' administrative areas where CPY (48% a.i. or similar concentrations) is commercialised and/or used for crops. Note: This map was built using Geographic Information Systems ArcGIS software, version 9.3 (Esri Inc., 2010;www.esri.com).

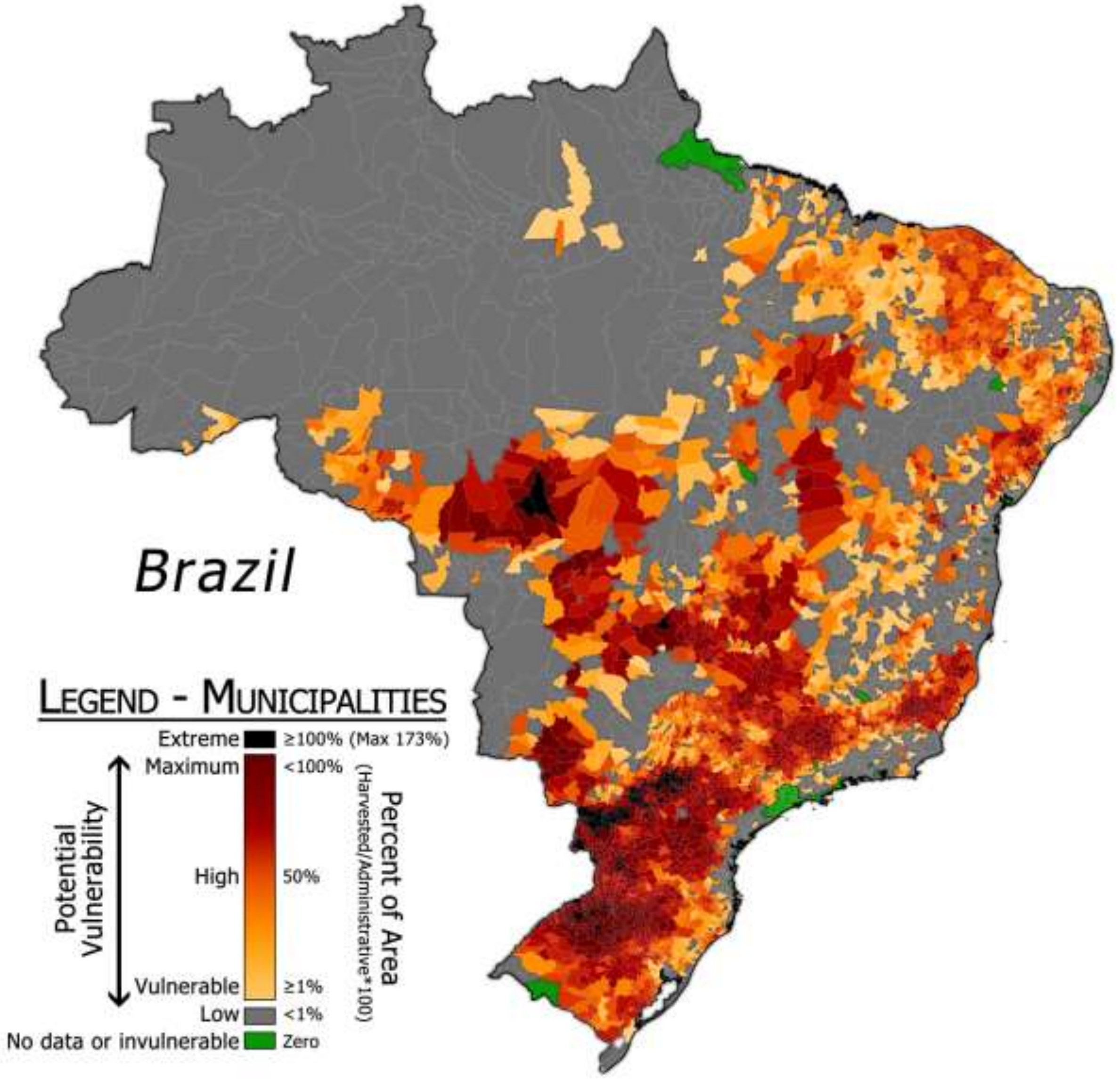

Supplementary Figure S3. Potential vulnerability of Brazilian stingless bee populations to chlorpyrifos (CPY). Brazilian municipalities' administrative areas where Lorsban® (480BR, 48% a.i., Dow AgroSciences, Brazil) is indicated for crops mentioned in the Methods, sub-item Brazil scale map. Note: This map was built using Geographic Information Systems ArcGIS software, version 9.3 (Esri Inc., 2010; www.esri.com). Note: The sum of the harvested area per year at some municipalities exceeds its administrative area because some crops are harvested more than once a year.

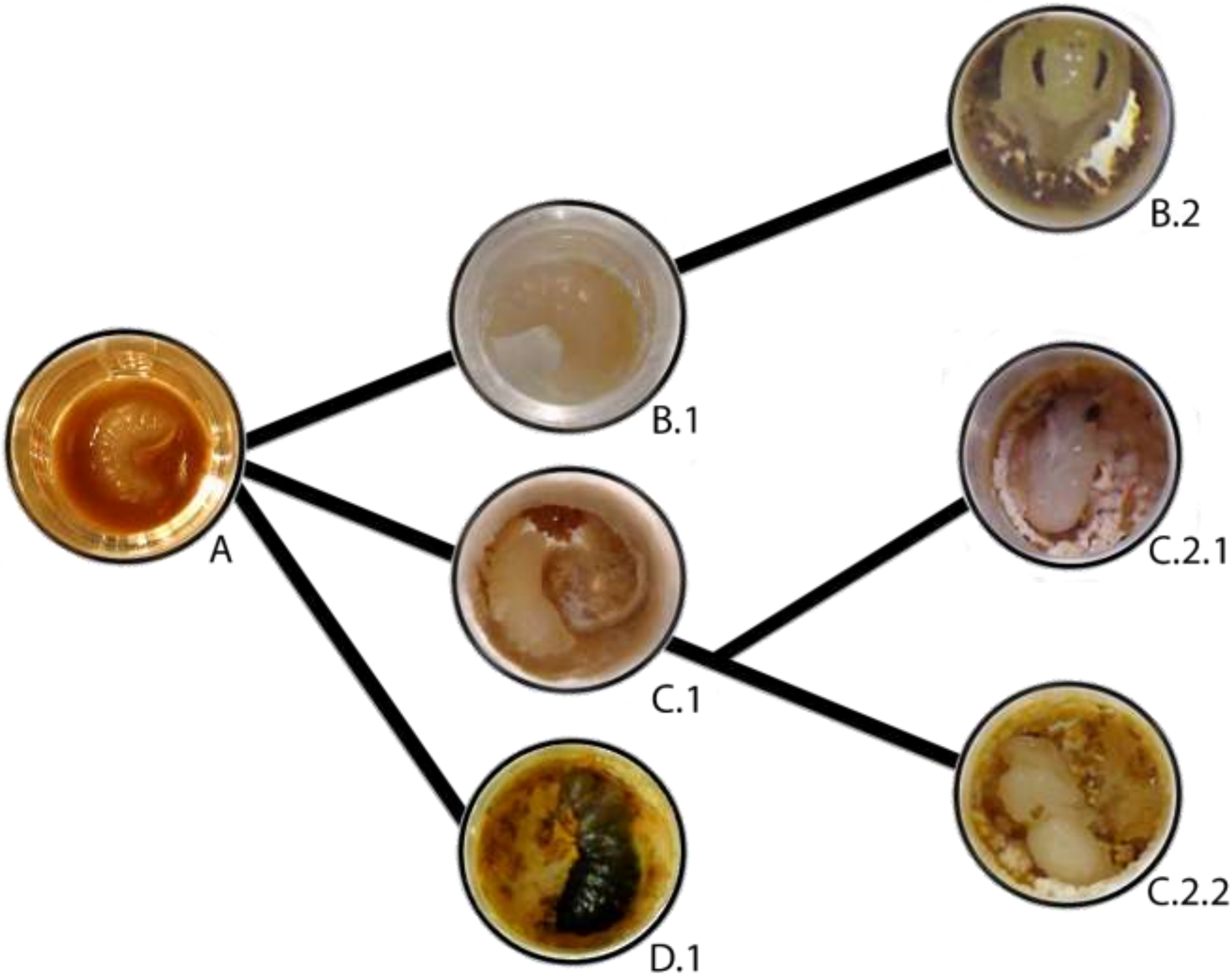

Supplementary Figure S4. Lethal and sublethal effects of chlorpyrifos. A – Feeding larva on liquid food; B.1 – Potential queen larva with nearly all food consumed; B.2 – Queen pupa surrounded by faeces; C.1 – Potential worker larva surrounded by faeces and waste food; C.2.1 – Worker pupa with ca. ⅔ of larval food consumed; C.2.2 – Worker pupa with ca. ⅓ of larval food consumed; D.1 – Dead pupa with largely darkened abdominal region. Images from author's archive.

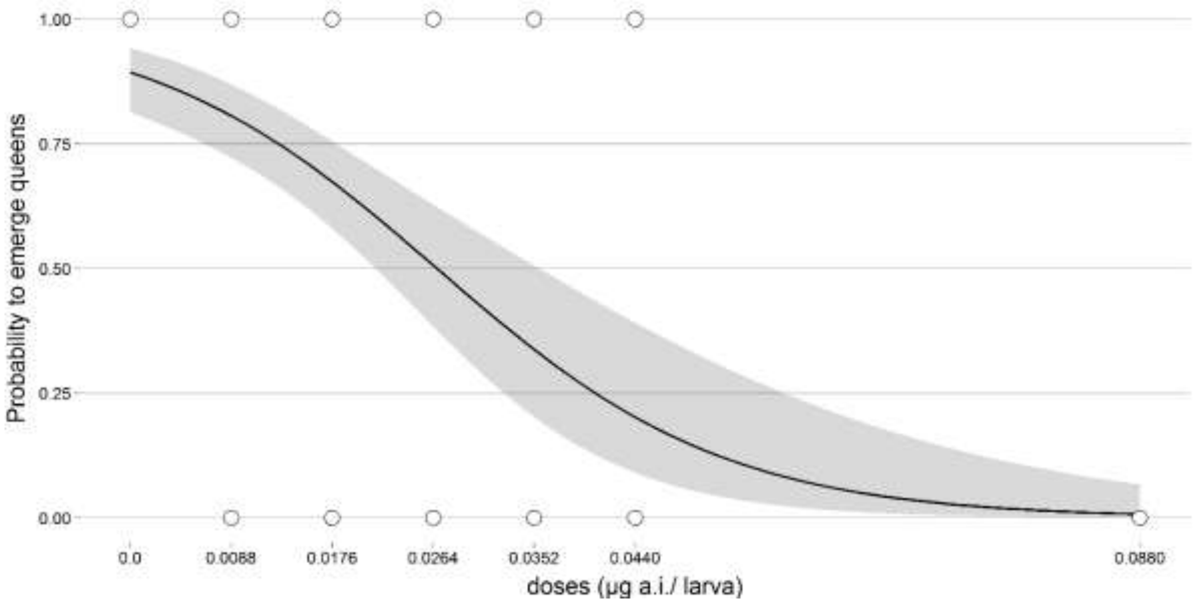

Supplementary Figure S5. Probability of emergence of queens. The chances of *Plebeia droryana* larvae to differentiate into a queen as a function of the dose of chlorpyrifos (CPY) in larval food. The binary logistic regression model (logit link) is shown. Legend: ○, observed data; —, the predicted model; shaded area, 95% confidence interval (generalised linear mixed model [GLMM] Binomial, P = 0.009).

Posterior legs of workers and queens

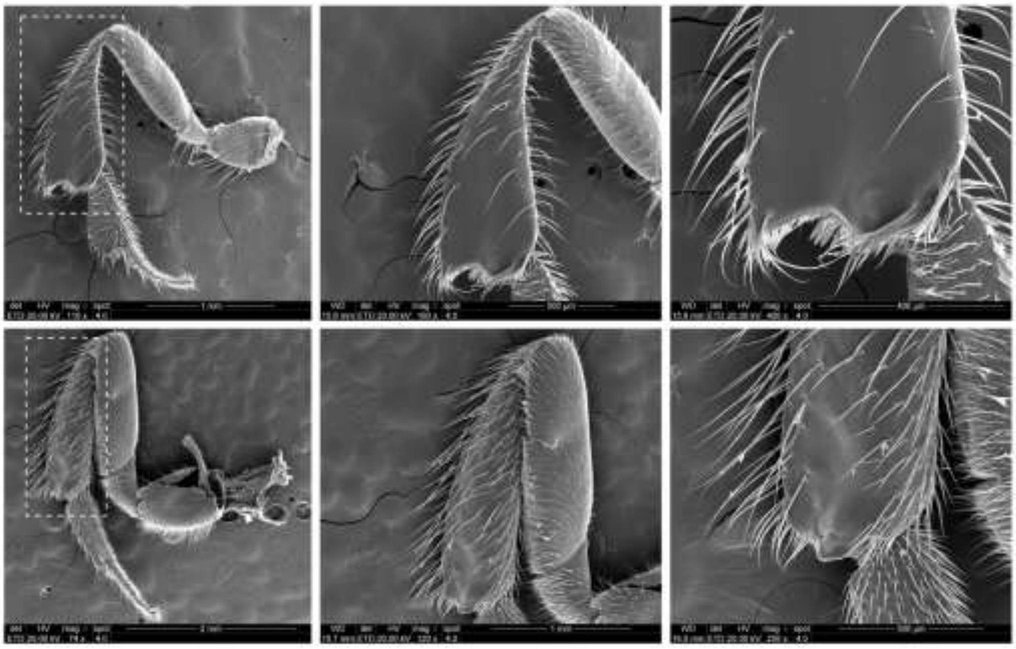

Supplementary Figure S6. Scanning electron microscopy of the posterior legs of *Plebeia droryana* workers and queens. Workers have wider and convex tibiae (top) that help them to carry pollen grains, resin, and other materials, whereas queens (bottom) lack this structure.

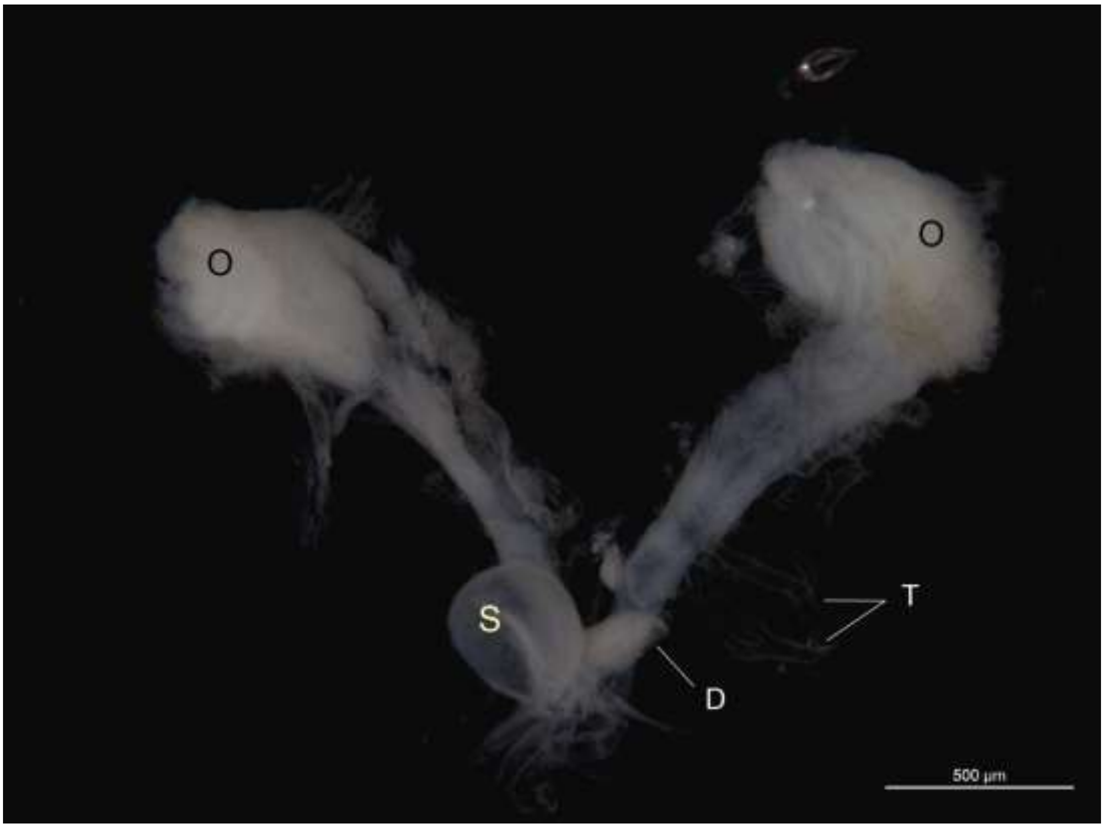

Supplementary Figure S7. Reproductive tract of *Plebeia droryana* **queens.** Legend: O, ovaries; T, Trachea; S, Spermatheca; D, Dufour´s gland. Note: Worker bees do not have well-developed ovaries neither they possess spermathecal reservoir.

Supplementary references

S1. Thompson, H. M., Wilkins, S., Battersby, A. H., Waite, R. J. & Wilkinson, D. The effects of four insect growth-regulating (IGR) insecticides on honeybee (*Apis mellifera* L.) colony development, queen rearing and drone sperm production. *Ecotoxicology* **14,** 757–769 (2005).

S2. Gregorc, A. & Ellis, J. D. Cell death localization in situ in laboratory reared honey bee (*Apis mellifera* L.) larvae treated with pesticides. *Pestic. Biochem. Physiol.* **99,** 200–207 (2011).

S3. Sandrock, C. *et al.* Impact of chronic neonicotinoid exposure on honeybee colony performance and queen supersedure. *PLoS One* **9,** e103592 (2014).

S4. DeGrandi-Hoffman, G., Chen, Y. & Simonds, R. The effects of pesticides on queen rearing and virus titers in honey bees (*Apis mellifera* L.). *Insects* **4,** 71–89 (2013).

S5. Whitehorn, P. R., O'Connor, S., Wackers, F. L. & Goulson, D. Neonicotinoid pesticide reduces bumble bee colony growth and queen production. *Science* **336,** 351–352 (2012).

S6. Laycock, I. & Cresswell, J. E. Repression and recuperation of brood production in *Bombus terrestris* bumble bees exposed to a pulse of the neonicotinoid pesticide imidacloprid. *PLoS One* **8,** e79872 (2013).

S7. Fauser-Misslin, A., Sadd, B. M., Neumann, P. & Sandrock, C. Influence of combined pesticide and parasite exposure on bumblebee colony traits in the laboratory. *J. Appl. Ecol.* **51,** 450–459 (2014).

S8. Scholer, J. & Krischik, V. Chronic exposure of imidacloprid and clothianidin reduce queen survival, foraging, and nectar storing in colonies of *Bombus impatiens*. *PLoS One* **9,** e91573 (2014).

S9. Bernauer, O., Gaines-Day, H. & Steffan, S. Colonies of bumble bees (*Bombus impatiens*) produce fewer workers, less bee biomass, and have smaller mother queens following fungicide exposure. *Insects* **6,** 478–488 (2015).

S10. Johnson, R. M., Ellis, M. D., Mullin, C. A. & Frazier, M. Pesticides and honey bee toxicity – USA. *Apidologie* **41,** 312–331 (2010).

S11. Mullin, C. A. *et al.* High levels of miticides and agrochemicals in North American apiaries: implications for honey bee health. *PLoS One* **5,** e9754 (2010).

S12. Al Naggar, Y. *et al.* Organophosphorus insecticides in honey, pollen and bees (*Apis mellifera* L.) and their potential hazard to bee colonies in Egypt. *Ecotoxicol. Environ. Saf.* **114,** 1–8 (2015).

S13. Bernal, J. *et al.* Overview of pesticide residues in stored pollen and their potential effect on bee colony (*Apis mellifera*) losses in Spain. *J. Econ. Entomol.* **103,** 1964–1971 (2010).

S14. Chauzat, M. P. *et al.* An assessment of honeybee colony matrices, *Apis mellifera* (Hymenoptera: Apidae) to monitor pesticide presence in continental France. *Environ. Toxicol. Chem.* **30,** 103–111 (2011).

S15. Pettis, J. S. *et al.* Crop pollination exposes honey bees to pesticides which alters their susceptibility to the gut pathogen *Nosema ceranae*. *PLoS One* **8,** e70182 (2013).

S16. Stoner, K. A. & Eitzer, B. D. Using a hazard quotient to evaluate pesticide residues detected in pollen trapped from honey bees (*Apis mellifera*) in Connecticut. *PLoS One* **8,** e77550 (2013).

S17. Pareja, L. *et al.* Detection of pesticides in active and depopulated beehives in uruguay. *Int. J. Environ. Res. Public Health* **8,** 3844–3858 (2011).

S18. Chauzat, M.-P. & Faucon, J.-P. Pesticide residues in beeswax samples collected from honey bee colonies (*Apis mellifera* L.) in France. *Pest Manag. Sci.* **63,** 1100–1106 (2007).

S19. Wu, J. Y., Anelli, C. M. & Sheppard, W. S. Sub-lethal effects of pesticide residues in brood comb on worker honey bee (*Apis mellifera*) development and longevity. *PLoS One* **6,** e14720 (2011).
